# Understanding the Supersensitive Anti-Drug Antibody Assay: Unexpected High Anti-Drug Antibody Incidence and Its Clinical Relevance

**DOI:** 10.1155/2016/3072586

**Published:** 2016-05-31

**Authors:** Sam Song, Lili Yang, William L. Trepicchio, Timothy Wyant

**Affiliations:** Immunogenicity Group, Department of Translational Medicine, Takeda Pharmaceutical USA, Inc., 35 Landsdowne Street, Cambridge, MA 02139, USA

## Abstract

Numbers of biotherapeutic products in development have increased over past decade. Despite providing significant benefits to patients with unmet needs, almost all protein-based biotherapeutics could induce unwanted immunogenicity, which result in a loss of efficacy and/or increase the risk of adverse reactions, such as infusion reactions, anaphylaxis, and even life-threatening response to endogenous proteins. Recognizing these possibilities, regulatory agencies request that immunogenicity be assessed as part of the approval process for biotherapeutics. Great efforts have been made to reduce drug immunogenicity through protein engineering. Accordingly the immunogenicity incidence has been reduced from around 80% in murine derived products to 0–10% in fully human products. However, recent improvements in immunogenicity assays have led to unexpectedly high immunogenicity rates, even in fully human products, leading to new challenges in assessing immunogenicity and its clinical relevance. These new immunogenicity assays are becoming supersensitive and able to detect more of anti-drug antibodies (ADA) than with earlier assays. This paper intends to review and discuss our understanding of the supersensitive ADA assay and the unexpected high ADA incidence and its potential clinical relevance.

## 1. Introduction

The approvals of the first recombinant human protein (insulin, 1982) and the first therapeutic monoclonal antibody (Muromonab-CD3, OKT3, 1985) symbolized the start of the new biotherapeutic era. Since then, development of biotherapeutics (i.e., biologics, biopharmaceuticals, biological products, and biological medicinal products/drugs) has increased. Today, more than 250 approved biotherapeutics are available for unmet medical needs and there are estimated >500 biotherapeutics at various stages of development [1, 2].

One of the major differences between large molecule-based biotherapeutics and traditional small molecule drugs is the potential of biotherapeutics to produce unwanted immunogenicity. The patient's immune system recognizes the administered biotherapeutic as foreign and produces ADA against the foreign molecule. While new technologies have greatly reduced the primary sequence of the protein to be essentially human the immune system can still recognize differences due to both the production lines (i.e., grown in NS0, CHO, or insect cell lines) which may lead to alterations in secondary and tertiary structures (i.e., glycosylation patterns and protein misfolding) and formulation as foreign. In case of therapeutic fusion protein, bispecific antibody, PEGylated antibody and antibody drug conjugate (ADC), and so forth the region where the two molecules join may form a neoantigen (foreignness), and immune responses to this region may arise. In addition, the regions in which these proteins are added (i.e., subcutaneous or intramuscular) also increase the foreignness of the protein. Although a cellular immune response is also involved in drug immunogenicity, the regulatory agencies usually do not request assessment of cellular immunogenicity as the formation of class switched antibodies is highly dependent on the cellular immune response. Therefore this review will not cover cellular immunogenicity.

Almost all protein-based biotherapeutics have the potential to induce immunogenicity and large efforts have been made to reduce drug immunogenicity, particularly in the therapeutic monoclonal antibody (mAb) field. One result of these efforts has been to facilitate the transition from murine and chimeric mAb to humanized and fully human monoclonal antibodies. Accordingly, the 84% ADA positive rate for murine products was reduced to 40% for chimeric products, to 9% for humanized products, and to 0–12% for fully human mAbs [1, 3–10]. Although it is inappropriate to compare the ADA positive rate between different products and different assays, the cited ADA incidences serve to illustrate the downward trend of ADA incidence that has occurred with the increased progress in therapeutic protein engineering.

An ADA response can result in a loss of efficacy and/or increase the risk of adverse reactions (e.g., infusion reactions, anaphylaxis, or immune-complex-mediated diseases) [3, 11–13]. In rare cases, the ADA response is directed not only to the administrated biotherapeutic but also to its endogenous counterpart protein and may elicit a life-threatening response in particular if the endogenous protein is unique and nonredundant and has a vital life function [6, 14]. In recognition of these possibilities, the regulatory agencies request that a biotherapeutic's immunogenicity be assessed and a determination of its characteristics relative to any induced clinical consequences be done as part of the approval process for biotherapeutics.

Without an appropriate immunogenicity assessment data package for a biotherapeutic, Biologic License Application (BLA) or New Drug Application (NDA) will have a much more difficult time being approved by the regulatory agencies. The immunogenicity assessment, in turn, highly depends on appropriate immunogenicity assays. In the past decade, drug developers, medical device industries, academics, and regulatory agencies have worked together to provide more technology platforms, different assay formats, and multiple important white papers [15, 16] and regulatory guidance (US Food and Drug Administration (FDA) and European Medicines Agency (EMA)) [17–21] that improved the ways to assess immunogenicity. Because some of our assays are now supersensitive and have better drug tolerance, they are able to detect more ADA, which has resulted in sharp increases in ADA positive rates, thereby generating new challenges and new issues for the drug developer. When a high ADA positive rate is unexpectedly seen, it should be asked as to why the ADA positive rate is so high and if the development of the drug should be put on hold. The regulatory agencies may ask for additional testing and characterization to ensure patient safety. Physicians may simply choose another drug with a lower ADA positive rate when there is more than one drug on the market for the same or similar indications, which would impact drug sales. This paper intends to review and understand the supersensitive ADA assay and examine the unexpected high ADA positive incidence and the potential clinical relevance of a high ADA positive incidence.

## 2. The Supersensitive ADA Assay

The sensitivity of the anti-drug antibody (ADA) assay is defined as the lowest concentration of ADA the assay method can reliably differentiate from background or the level of ADA response that is equal to or above the assay cut point (CP). The original FDA draft guidance has recommended the ADA screening assay sensitivity be around 250–500 ng/mL to be able to pick up clinically relevant immunogenicity; however, recent FDA draft guidance has now lowered this to 100 ng/mL as they have observed clinically relevant responses at this level [17, 21]. A supersensitive ADA assay is defined as an assay that is able to detect single to low double digit ng per mL of ADA in the testing sample. The development of supersensitive ADA assays has been possible because of better technologies and more experience in assay development and validation.

To evaluate the ADA sensitivity, we must know the assay drug tolerance limit. Without knowing the drug tolerance limit, the assay sensitivity is almost meaningless because biotherapeutics usually have a long half-life and are almost always present in the testing sample at most sampling time points. If the ADA assay drug tolerance level is lower than the drug concentration in the testing samples, the ADA assay is not able to detect ADA present in the sample due to interference of the drug and the ADA incidence would be underestimated. Although the regulatory guidance does not recommend an acceptable drug tolerance level, ideally we want the ADA assay to be able to tolerate higher than the trough level of the drug as ADA samples are most often taken during the drug trough period.

Unlike a quantitative assay that utilizes an appropriate reference standard curve to differentiate a positive response from background noise and to calculate the analyte concentrations in the study samples, there is no reference standard available in an ADA assay. The ADA assay is a qualitative or quasi-quantitative assay in which the CP is applied to differentiate ADA positives samples from negative samples and the surrogate positive control (PC) generated from immunized animals is used to assess the ADA assay sensitivity. The surrogate positive control cannot be expected to represent the spectrum of immune responses observed in individuals treated with the drug in clinical studies and the actual assay sensitivity and the drug tolerance level may also be different in the clinical sample testing. However, using the surrogate ADA positive control from immunized animals is considered to be the best practice in the industry and is accepted by regulatory agencies (FDA, EMA, etc.).

Previously, enzyme linked immunosorbent assay (ELISA) methods were used in most ADA assays and were associated with a higher background noise that resulted in a higher minimum required dilution (MRD) and lower assay sensitivity. It has also been reported that the drug tolerance levels in some of the earlier ADA assays were lower than the drug concentration present in the clinical testing samples [22]. Wang et al. [22] reviewed 28 FDA-approved biotherapeutics (10 proteins, 2 Fab products, 4 Fc fusion proteins, and 12 monoclonal antibodies) during 2005–2011 and found many FDA-approved biotherapeutics had higher steady-state drug concentrations than the drug tolerance of ADA assays by 1.2- to 800-fold. Consequently, the reported immunogenicity rates for the 28 biotherapeutics may have been underestimated.

Their survey showed that drug tolerance of the ADA assay for 19 products spanned the range between 1 ng/mL and 50 *μ*g/mL whereas the steady-state trough drug concentrations of 22 products ranged from 0.3 ng/mL to nearly 400 *μ*g/mL. They found that the ADA assays of more than half of approved products (13 out of 22 with appropriate data for evaluation) had a drug tolerance level lower than the steady-state trough drug concentration. These consisted of 9 out of 12 monoclonal antibody products, 2 out of 4 Fc fusion protein products, 1 out of 10 protein products, and 1 out of 2 Fab products, which suggested that the ADA assays for monoclonal antibody molecules were more susceptible to drug interference. Interestingly, many FDA-approved monoclonal antibody products (12 products as of March 2012) had postmarketing requirements/commitments (PMR/PMC) to develop improved immunogenicity assays and to assess the impact of immunogenicity after the new and improved assays were implemented.

For the monoclonal antibody biotherapeutic Humira (adalimumab), it was reported that the drug tolerance level for the anti-adalimumab antibody assay was less than 2 *μ*g/mL, while the adalimumab trough levels were 5 *μ*g/mL and 8 to 9 *μ*g/mL. According to the drug insert, the ADA positive rate for rheumatoid arthritis (RA) patients is 5% [23], which might be underestimated due to the drug tolerance issue. In the plaque psoriasis clinical study, only approximately 40% of subjects had less than 2 *μ*g/mL of adalimumab in their ADA testing samples.

In the past decade many new technologies have become available to detect immunogenicity, such as Meso Scale Discovery (MSD) electrochemiluminescence (ECL), Gyros, ImmunoCAP, and SQI Diagnostics. In addition, new sample pretreatment approaches, such as acid dissociation and SPEAD, to improve drug tolerance have also been developed and incorporated into the new generation of ADA assays [3, 6, 7, 24–32]. These methodology advances have increased assay sensitivity dramatically. Today, most ADA assays are ECL technology with bridging format and acid dissociation, which has made the assay background clean (less noise), the drug tolerance better, and the assay more sensitive. With more sensitive or supersensitive ADA assays, a higher ADA incidence is expected. However, if the new ADA positive rate is much higher (e.g., a 5–10-fold increase) than previous ADA reports or historical ADA reports or expected rate, it becomes imperative to interpret and report the results based on clinical relevance rather than total positive rates [33].

## 3. Are All ADA Positives True Positives?

When a high ADA positive rate is unexpectedly observed, first, it should be asked if all of the ADA positives are true positives. Does being equal to or just above the screening assay and confirmatory cut points mean they are a true positive? The following questions must be answered before accepting that the positives are true positives. Were the screening and confirmatory cut points set correctly? If outliers were removed, is it certain that the data point removed is not the part of the study population and the data still accurately reflects the biological variability? Was enough of the normal range of biological and analytical background noise included into the screening CP and confirmatory CP to ensure the CPs are not set too low? Does the drug have a soluble target in the clinical testing sample? If it does, has it been determined during assay development and validation if the soluble drug target has a positive interference on the assay? If it does have a positive interference, has an approach to remove the interference in the assay been developed? All of these questions should be answered during assay development and assay validation. However, sometimes the well-validated assay in prestudy phase does not work as expected during the study phase. This may occur as a result of the following: (1) in the prestudy stage (when assay development and validation are done), the CP is determined with commercially available serum lots, either normal healthy or disease state serum lots, that might not represent the serum of the study population and the CP may not be appropriate; (2) the assay developer lacks knowledge about the drug's mechanism of action (MOA) and the assay format is not chosen appropriately; (3) potential interference factors presented in the testing samples are not investigated thoroughly during assay development; (4) statistical tools are not appropriately used in the CP determination (e.g., too many outliers might be removed); (5) during the assay development, the interrelations of assay sensitivity, drug tolerance, and CP are not balanced appropriately [15]; and (6) the assay background noise is so low that the response of drug naive serum is near or at the relative lower limits of the instruments. In the following section, examples will be discussed in detail to explain how these factors impact the ADA positive rate.

### 3.1. Drug Soluble Target May Cause False High Positive ADA Rate

It has been well accepted that the bridging ADA screening ECL assay with acid dissociation is one of the most popular and effective ADA screening assays. In this assay format, a biotin-labeled drug is used as capture and a Sulfo-TAG*™*-labeled drug is used as detection. ADA present in the testing sample can be detected because ADA can bridge both the labeled capture and the detection drug. The advantages of the bridging ADA screening assay are that it is species independent, it is able to detect all isotypes (except IgG4 due to Fab arm exchange), and it is easy and convenient. The weakness of this assay format is that it is prone to interference by free drug in the testing sample, which may cause a false negative [30]. To overcome the drug interference, acid dissociation and/or drug removing approaches have been incorporated into the bridging assay format, allowing ADA to be successfully detected in the presence of excess free drug in samples, and have almost become the universal approach in preclinical and clinical ADA screening [26, 27, 31].

However, due to the fact that drug conjugates are utilized as the capture and detection molecules, this type of approach is also susceptible to soluble drug target interference. Bivalent soluble drug target (homodimer or multimer) can produce a false positive signal due to its ability to bridge the labeled capture and detection drugs in the assay system. In the ADA testing samples, the soluble drug target or ligand forms complexes with the drug while soluble drug forms complex with ADA. The acid dissociation not only dissociates ADA from the drug/ADA complex but will also dissociate drug target from the drug/drug target complexes to make more drug target available. If the soluble drug target is bivalent, it could bridge the labeled drugs in the assay system and produce a false ADA positive result. If monovalent, soluble drug targets could also cause a false negative result.

Dai et al. [31] recently reported an unexpected high ADA incidence (>60%) in fulranumab phase I clinical study samples using the bridging ADA screening assay format with acid dissociation. Fulranumab is a human IgG2 monoclonal antibody that neutralizes nerve growth factor (NGF) and currently it is in development for the treatment of pain. They speculated that NGF produced a false positive signal because of its ability to bridge fulranumab molecules in their assay system. Thus, they then revised the assay, which involved a two-step specificity confirmation assay that first used anti-NGF antibody-coated beads to selectively remove NGF (both free and bound to fulranumab), followed by competitive inhibition with fulranumab. Using the revised method, they confirmed that the high apparent anti-fulranumab antibody incidence (>60%) in the clinical study samples was in fact due to fulranumab-bound NGF released during the acid dissociation step of the ADA testing method. Analysis of samples from four phase 2 clinical studies showed that ADA positive rates were >50% in the screening assay but only 1.3% or less were confirmed true positives using their “double” confirmatory assay. The majority of the false ADA positives were because of NGF positive interference.

Zou [32] also observed an unexpected high ADA incidence (>70%) in a phase I clinical study of a therapeutic humanized antibody (TA), which had a soluble target (ST) present in the serum. After investigation, it turned out that the majority (~90%) of TA-confirmed samples were proved to be false positive due to ST interference. They developed a double competitive confirmatory assay, with which they were able to eliminate false positives caused by ST and were able to identify a true ADA positive of only 6.6%–7.8%.

It is recommended that when soluble targets are found in the testing sample as a divalent molecule or potentially forming a divalent molecule (dimer or multimer), the drug target interference be assessed during assay development and an appropriate approach to overcome the drug target interference be developed and validated. Developing an immunogenicity assay without consideration of the MOA should be avoided. As the assay developer or assay scientist, it is essential to communicate to the R&D team early in the drug development phase and understand the MOA, which will help the analyst choose the appropriate assay technology platform, assay format, and reagents.

The ADA screening bridging assay format with acid dissociation should not be used as the universal approach in immunogenicity testing. For example, as stated above, when the drug has a divalent soluble target the acid pretreatment procedure included in the screening assay may make target interference worse by releasing additional target from drug/target immune complexes and may result in a higher false ADA positive rate. In the past, it was underappreciated that the drug target can cause a high false ADA positive in immunogenicity testing. This point should be kept in mind when ADA assays are developed and when the clinical ADA data are analyzed, particularly when the ADA positive rate is unexpectedly high.

### 3.2. Unnecessary Outlier Removal May Cause a Lower CP and a Higher ADA Positive Rate

Identification and removal of outliers is an important step in the CP determination because outliers can change the value of the CP. In most cases, the CP raw data do not result in a normal (bell shape) distribution; instead, the majority of data points are low values distributed to the left side of the mean, while high values are distributed on the right side of the mean and form a long tail (i.e., skewed to the right or a positive skew). The positive skew of ADA screening data is often a result of the values being at the low end of the instruments signal and therefore tending to “pile” at that end. Thus when considering outliers, the overall representative true variability must incorporate the variability caused by the sample and not just the instrument. In general these new assays have removed so much serum variability that often only the instrument and analytical variability are measured which may not be sufficient to capture patient variability in the clinical trial. Hence when considering outlier removal a highly conservative approach must be taken.

An outlier is a value that “lies outside” most of the other values in a set of data. As outliers, they can be either smaller or larger values and are distributed far from the major population. There are two kinds of outliers in an immunogenicity assay: biological outliers and analytical outliers. Generally, in immunogenicity data the values for outliers are much larger than those of the major population. Outliers should be removed during the CP determination. One of the most common statistical methods used to identify an outlier is the Outlier Box Plot.

**Figure 1:**
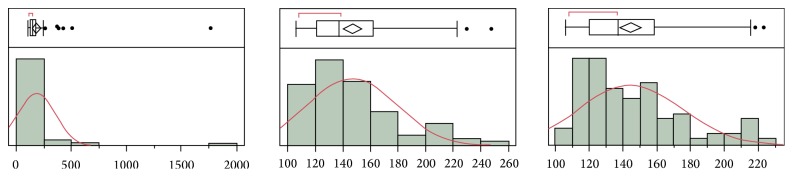
Outlier identification and distribution of the drug naïve matrix sample results using JMP Outlier Box Plot. The Outlier Box Plot is composed of the top and bottom parts. The bottom part is a histogram plot which indicates the data distribution. *x*-axis represents data values (assay response values) and *y*-axis represents data frequency. The red curve is the normal density curve. The top part is Quantile Box Plot (the Outlier Box Plot) and the disconnected points are potential outliers. A red bracket defines the shortest half of the data (the densest region). The results of the first, second, and third run of the outlier identification are displayed in each individual plot from left to right.

The case study given here shows the challenge to identify outliers and illustrates which outliers should be removed. The graph (Figure 1) shows box plot analysis of 100 lots of normal drug naïve individual serums in an actual CP experiment. Seven outliers were identified and they were removed in the first run of outlier identification, followed by a second run in which 2 more outliers were identified and removed. After the 9 outliers were removed, a third run identified 2 more outliers. Should we continue to remove all outliers identified by an Outlier Box Plot?

The outliers identified may make sense from a statistical point of view, but do they make sense from a biological point of view? Obviously solely relying on statistical analysis for this determination would not suffice and therefore the data must be judged biologically and statistically. In general, in the authors' experience, removing outliers after only one run works the best. If the data fit a normal distribution (Gaussian distribution) after the removal of the outliers identified in the first run, the CP can be calculated using a parametric approach. However, if the data do not fit a normal distribution, transform all the data, including “outliers,” to log scale and see if the data fit a log normal distribution with and without the outliers removal. If the transformed data fit a log normal distribution, either with or without the outliers, calculate the CP on log scale using a parametric approach. If the data do not fit a log normal distribution after one run of outlier removal, then calculate the CP with the nontransformed data using a nonparametric approach.

The second run of outlier removal could be considered only if the situation meets the following criteria: (1) the data point(s) are very far from the major data population; (2) only a few of the outliers were removed in the first run; and (3) there is a biological reason to believe they are outliers. It can simply be the case that some observations happen to be a long way from the center of the data even though the data belong to the major population. It has been recognized that data have biological significance even though identified as outliers using the box plot. Elimination of those biologically significant data points would bias the value of the CP and further bias the results of the study.

When data points can be identified as resulting from analytical mistakes (a measurement mistake or a technique mistake), then a case can be made for elimination of the data points from the data set because they do not represent the population/experiment in question. Anytime a single outliner is rejected we run the risk of throwing away biologically significant data.

Outlier identification and exclusion may not be straightforward and sometimes can be problematic dependent on the population being tested. For example, in a rheumatoid arthritis (RA) population, many outliers may be identified using a box plot analysis because RA serum contains rheumatoid factor as well as other unknown interfering agents, which can generate higher signals. However by excluding these in the analysis, which results in a lower CP, the majority of the testing samples will become positive for ADA thus making drug specific information more difficult to ascertain. Therefore it is suggested to be very cautious in removing outliers in populations such as the RA patient population.

Whenever an outlier is removed, it should be asked if there is a valid reason to remove it. We cannot simply remove all outliers identified only by the statistical tool. If there is no reason to remove them, then the points should be kept. It is critical to take a conservative approach to remove outliers to set up the CP properly, which in turn will impact the ADA positive rate. When there is a very high ADA positive rate, one question that should be asked is, were too many “outliers” removed and is it certain that the deleted outliers do not belong to the study population? Considering the ADA assay today has much less background noise and the assay background range is very narrow, it is recommended not to exclude too many data points as “outliers” unless we are certain they are outliers from both statistical and biological standpoint.

### 3.3. Inappropriate CP Established in Prestudy Stage May Cause High ADA Positive Rate in Study Stage

Usually the CP is established during the ADA assay validation in the prestudy stage using a commercially available serum matrix, which can be either normal or disease state serum. Whether the CP established in the prestudy stage works in the study stage is mainly dependent on whether the commercially available serum represents that of the study population. Unfortunately, sometimes the commercially available serum does not react in the same or similar way to the serum samples from the study population and can result in either a false high or low positive rate. Based on our experience, there is a higher probability to cause a false higher positive rate than that of a false lower positive rate, particularly when the ADA assay is supersensitive. If an ADA positive rate of more than 15% is seen in the baseline samples, it should trigger a CP reassessment and the need to reset the CP using the clinical baseline samples.

Currently, most assays are developed with a 5% false positive rate built into the screening CP to ensure capture of all true positives. While a target of 5% false positive in screening is the goal, often the actual false positive rate for a screening CP is either lower or higher than 5%. In our experience, the screening CP was more often higher than 5% and up to 10% or above. The 2015 9th Workshop on Recent Issues in Bioanalysis (WRIB) recommended [34] that if the false positive rate of the in-study baseline samples is too low (<2%) or too high (>11%), the means and variances of the log-transformed ratio of individual subject sera to negative control from the validation (prestudy) and clinical study baseline (in-study) be compared first. If only the means of these ratios are significantly different, use the variance from the validation along with the mean of the ratios from the in-study baseline samples to adjust the CP factor accordingly. If the variances are different, the in-study baseline samples may be used to calculate a new study-specific CP correction factor. This is reasonable to do as long as baseline data are available from at least 50 subjects, tested over at least two runs and by at least two analysts. More detailed information about the study-specific CP reassessment is available in [16, 34].

## 4. Unexpected High ADA Positive Rate and Its Clinical Relevance

The majority of high ADA positive rates are caused by the supersensitive ADA assay which is able to detect more ADA than the earlier assays. After ruling out a false high ADA positive due to any positive interferences or technical issues such as the CP setup issues, the high ADA positive rate must be accepted and appropriately interpreted to know what the clinical relevance of the high ADA positive rate means. It should be noted that clinical efficacy and adverse effects may not change much despite a dramatically increased ADA positive rate. For example, the ADA positive rate of Tysabri increased from 4.5% to 58%, but only 25% out of the 58% of the patients with high ADA titers also had adverse effects and a loss in drug efficacy [35]. There are numerous reports which indicate that persistent ADA positives rates with high titers are closely related to adverse effects and a loss in clinical efficacy, while transient ADA positives rates with low titer often have little or no loss of clinical efficacy or safety issues [3, 11, 12, 33, 35–38]. Therefore it is very important to break down the total ADA positive population into ADA positive subpopulations to identify which subpopulation of ADA positive is clinically relevant. The clinically relevant ADA is defined as the ADA which impacts safety, efficacy, or pharmacokinetics regardless of persistence or transience or titer level. However, often the clinical relevant ADA is associated with the persistent ADA with moderate to high titers. On the other hand, the nonclinically relevant ADA is the ADA which has no or little impact on safety, efficacy, or pharmacokinetics regardless of persistence or transience or titer level, but the nonclinically relevant ADA often is associated with the transient ADA and low titer. It is speculated that a dramatic increase in ADA positive rate is mainly because of an increase in the nonclinical relevant ADA fraction, which usually occurs in a low assay signal. This kind of low assay signal is easily picked up by the supersensitive ADA assay, while it was often missed by the older ADA assays used previously.

In 2013, Vennegoor et al. reported [35] on the clinical relevance of serum natalizumab (Tysabri) concentration and anti-natalizumab antibodies in multiple sclerosis. Their results showed that patients with a high antibody titer had a 10.5 times higher odds ratio (OR) (*p* = 0.02) to develop gadolinium positive (Gd+) lesions and 10.9 times higher odds (*p* = 0.008) to have a relapse compared to patients with no antibodies. They found the low concentration drug was associated with high titer of ADA and had a 14.5 times higher OR (*p* = 0.006) to develop gadolinium positive Gd+ lesions and a nine times higher OR (*p* = 0.01) to have a relapse compared to normal serum natalizumab concentrations.

Their data showed that only 25% of all patients had high antibody titers (range 110–260,000 AU/mL) at least at one time point during the study and demonstrated the clinical relevance of the persistent ADA with high titer. The authors did not state the clinical relevance of transient ADA with low titer; however, it was noted that patients with low antibody titer had similar Gd+ lesions as those with no antibody titer. Presumably, the low antibody titers, possibly in combination with a relatively low affinity, were insufficient to significantly affect natalizumab concentrations. They concluded that both low natalizumab serum concentration and high antibody titers were associated with a lack of efficacy of natalizumab.

Interestingly, the authors found a substantially higher percentage (58%) of patients with anti-natalizumab antibody positive results than had been previously reported (58% versus 4.5–14.1%) [36–39]. They postulated the difference was most likely due to the differences in the assay methods used. The RIA method they used was better suited than the ELISA method to detect anti-natalizumab antibodies in serum when free natalizumab is also present in the serum [35]. In addition, the possibility that the ELISA assay did not have as good drug tolerance capability as the newer ADA assay also may have been a factor.

The majority of the reports on the clinical relevance of ADA incidence are short-term studies that show the development of ADA is associated with diminished drug serum levels and a diminished treatment response. However, little is known about the clinical relevance of ADA against these drugs during a long-term follow-up.

Baert et al. [11] reported the influence of immunogenicity on the long-term efficacy of infliximab in Crohn's disease. Infliximab (Remicade), used to treat autoimmune diseases, is a chimeric monoclonal IgG1 antibody against tumor necrosis factor. In a cohort of 125 consecutive patients with Crohn's disease treated with infliximab infusions, the authors evaluated the concentrations of infliximab and of antibodies against infliximab, clinical data, and side effects (including infusion reactions) before and 4, 8, and 12 weeks after each infusion. A mean of 3.9 infusions (range, 1 to 17) per patient were administered over a mean period of 10 months. The median follow-up was 36 months (range, 25 to 48). After the fifth infusion, 76 patients (61%) had detectable antibodies and the incidence did not increase further with repeated infusions. But only 46 out of the 125 patients (37%) had a high titer of ADA (the author used 8.0 *μ*g ADA per milliliter as the threshold to differentiate high titer of ADA from low titer of ADA (see [12] for the detailed information)). The presence of high titer ADA before an infusion predicted a shorter duration of response (35 days, as compared with 71 days among patients with low titer of ADA (*p* < 0.001) and a higher risk of infusion reactions (*p* < 0.001) and a lower concentration of infliximab). Based on their results, the authors concluded that the development of high titer antibodies against infliximab was associated with an increased risk of infusion reactions and a reduced duration of response to treatment.

Bartelds et al. [12] reported the development of anti-drug antibodies against adalimumab (Humira) and its clinical relevance during a 3-year follow-up in 272 patients with rheumatoid arthritis (RA). In their study, the CP was set as the ADA concentration that exceeded 12 AU/mL (AU, arbitrary units, 1 AU = 12 ng/mL ADA) and the adalimumab concentration was 5 mg/L or less. The mean cutoff value was derived from 100 healthy donors and set at 12 AU/mL. They divided the patients with ADA positives into two groups: low ADA titer group (≤100 AU/mL) and high ADA titer group (>100 AU/mL).

After 3 years, 76 of 272 patients (28%) developed anti-adalimumab antibodies (ADA). Patients without ADA had much higher adalimumab concentrations compared with patients with ADA. Forty-five of 76 patients (72.4%) had low ADA titer at all time points and 31 patients (27.6%) had high ADA titer at one or more time points. The median adalimumab concentration for patients without antibodies was 12 mg/L, for patients with low ADA titers median adalimumab concentration was 5 mg/L, and for patients with high ADA titer median adalimumab concentration was 0–3 mg/L. However, among the total ADA positive group, only 27.6% patients, who had high ADA titer, had very low drug concentration, lower minimal disease activity, and less often achieved remission. Their data showed the majority (72.4%) of the ADA positive were low titer and only the minority of ADA positive population (27.6%), who had high ADA titer, had obvious lower adalimumab concentration and lower minimal disease activity or clinical remission.

## 5. Conclusions

The very high ADA positive rate seen in recent reports relative to the low ADA positive rate seen in earlier reports suggests the ADA positive rate in earlier studies may have been underestimated (false negative) due to the low assay sensitivity and low drug tolerance. In contrast, the supersensitive ADA assay is able to pick up a low assay signal very close to the assay background noise and while it is usually a nonclinically relevant ADA, it may be a major contributor to the high ADA positive rate. In addition, the positive interference that can result from free drug target interference may also contribute to a very high ADA positive rate (false positive) in some of the cases. False negative and false positive data resulting from inaccurate test methods can lead to flawed correlations of ADA with clinical safety, pharmacokinetics, and efficacy results. It is, therefore, recommended that the first step of analysis and interpretation of ADA data when an unexpectedly very high ADA rate is observed is to rule out possible positive or negative interference and to check the appropriateness of the CP setup and then interpret ADA data in context with PK/PD, efficacy, and safety profiles. It has been reported that high titer of persistent ADA is usually associated with safety issues and the loss of efficacy in the clinic, while low titer of transient ADA usually has no or little clinical impact.

Improvement of bioanalytical methods and advancement of technology platforms that continue through the life cycles of drug development and the bioanalytical method itself not only benefit drug development, but eventually benefit the patients as well. With supersensitive ADA assays, we are able to detect ADA not previously detected by the earlier ADA assays and this level of sensitivity not only helps us better understand the drug immunogenicity risk, but also helps ensure patient safety. However it must also be recognized that with supersensitive ADA assays the total ADA incidence may increase significantly and that the total ADA incidence might be misleading if the clinical relevance is not clear. It is necessary to dissect the ADA positive population to identify the clinical relevant ADA positive subpopulation in order to provide clinically meaningful immunogenicity information to the physicians so they know how to assess and manage immunogenicity in the clinic.
